# Supplementary material for: Structural Development of Speech Networks in Young Children: A Cross-Sectional Study
Source: Neurobiol Lang (Camb). 2025 Jun 18;6:nol_a_00168. doi: 10.1162/nol_a_00168 (PMC12204736; doi:10.1162/nol_a_00168)
Supplement: Supplementary file 1 [file nol-6-1-168-s001.pdf]

0.1 | Supplementary Tables

Effects of Age and ADHD Symptomology for HNT

Tract	Coefficient	Estimate (Std Error)	t-value
Left Frontal Aslant Tract	Age	-0.021 (0.003)	-6.776***
	Mean Hyperactivity	0.065 (0.005)	13.386***
	Mean Inattention	-0.038 (0.004)	-8.606***
Right Frontal Aslant Tract	Age	-0.046 (0.003)	-15.097***
	Mean Hyperactivity	0.027 (0.005)	5.217***
	Mean Inattention	0.014 (0.005)	3.142**
Left Arcuate Fasciculus	Age	-0.033 (0.003)	-9.480***
	Mean Hyperactivity	-0.046 (0.006)	-7.987***
	Mean Inattention	0.0216 (0.005)	4.251***
Right Arcuate Fasciculus	Age	-0.030 (0.003)	-10.041***
	Mean Hyperactivity	0.008 (0.005)	1.507
	Mean Inattention	-0.022 (0.004)	-5.058***
Left Superior Longitudinal Fasciculus III	Age	-0.013 (0.003)	-3.950***
	Mean Hyperactivity	0.067 (0.006)	11.937***
	Mean Inattention	-0.044 (0.005)	-8.803***
Right Superior Longitudinal Fasciculus III	Age	-0.035 (0.003)	-10.695***
	Mean Hyperactivity	0.042 (0.006)	7.628***
	Mean Inattention	-0.014 (0.005)	-2.966**
Left Superior Cerebellar Peduncle	Age	-0.042 (0.012)	-3.633***
	Mean Hyperactivity	0.109 (0.021)	5.306***
	Mean Inattention	-0.041 (0.020)	-2.008*
Right Superior Cerebellar Peduncle	Age	0.0154 (0.009)	1.686
	Mean Hyperactivity	0.002 (0.014)	0.169
	Mean Inattention	-0.004 (0.013)	-0.308
Left Middle Cerebellar Peduncle	Age	-0.034 (0.007)	-4.815***
	Mean Hyperactivity	-0.070 (0.012)	-5.923***
	Mean Inattention	0.053 (0.010)	5.008***
Right Middle Cerebellar Peduncle	Age	-0.013 (0.007)	-1.930
	Mean Hyperactivity	-0.030 (0.011)	-2.591**
	Mean Inattention	0.008 (0.010)	0.807
Left Inferior Cerebellar Peduncle	Age	-0.020 (0.005)	-4.383***
	Mean Hyperactivity	0.032 (0.008)	4.216***
	Mean Inattention	0.036 (0.007)	5.373***
Right Inferior Cerebellar Peduncle	Age	0.022 (0.008)	2.764**
	Mean Hyperactivity	0.002 (0.012)	0.139
	Mean Inattention	-0.033 (0.011)	-2.983**

Effects of Age and ADHD Symptomology for RNT

Tract	Coefficient	Estimate (Std Error)	t-value
Left Frontal Aslant Tract	Age	0.035 (0.003)	10.993***
	Mean Hyperactivity	-0.062 (0.005)	-11.922***
	Mean Inattention	0.033 (0.005)	6.922***
Right Frontal Aslant Tract	Age	0.041 (0.003)	13.456***
	Mean Hyperactivity	-0.041 (0.005)	-8.060***
	Mean Inattention	-0.001 (0.005)	-0.267
Left Arcuate Fasciculus	Age	0.039 (0.004)	8.873***
	Mean Hyperactivity	0.029 (0.007)	4.035***
	Mean Inattention	-0.014 (0.006)	-2.215*
Right Arcuate Fasciculus	Age	0.021 (0.004)	5.061***
	Mean Hyperactivity	-0.081 (0.007)	-11.745***
	Mean Inattention	0.074 (0.006)	11.911***
Left Superior Longitudinal Fasciculus III	Age	0.005 (0.004)	1.278
	Mean Hyperactivity	-0.092 (0.007)	-13.995***
	Mean Inattention	0.049 (0.006)	8.287***
Right Superior Longitudinal Fasciculus III	Age	0.069 (0.004)	15.978***
	Mean Hyperactivity	-0.105 (0.007)	-15.187***
	Mean Inattention	0.059 (0.006)	9.512***
Left Superior Cerebellar Peduncle	Age	0.024 (0.013)	1.887
	Mean Hyperactivity	-0.001 (0.022)	-0.005
	Mean Inattention	0.012 (0.022)	0.562
Right Superior Cerebellar Peduncle	Age	0.099 (0.012)	8.126***
	Mean Hyperactivity	0.04 (0.022)	1.793
	Mean Inattention	0.063 (0.017)	3.733***
Left Middle Cerebellar Peduncle	Age	-0.022 (0.010)	-2.205*
	Mean Hyperactivity	-0.095 (0.016)	-5.822***
	Mean Inattention	0.119 (0.015)	7.802***
Right Middle Cerebellar Peduncle	Age	0.095 (0.010)	9.786***
	Mean Hyperactivity	-0.003 (0.015)	-0.188
	Mean Inattention	-0.010 (0.014)	-0.686
Left Inferior Cerebellar Peduncle	Age	0.013 (0.004)	2.871**
	Mean Hyperactivity	-0.003 (0.007)	-0.371
	Mean Inattention	-0.005 (0.006)	-0.805
Right Inferior Cerebellar Peduncle	Age	0.033 (0.007)	4.896***
	Mean Hyperactivity	-0.055 (0.010)	-5.390***
	Mean Inattention	0.050 (0.010)	5.088***

TABLE 1 Table 1 provides an overview of the effects of age and ADHD symtomology for the hindered (HNT) and restricted (RNT) restriction spectrum imaging (RSI) components. Across most of the tracts, age, mean hyperactivity, and mean impulsivity were significant for both HNT and RNT. Notably, these coefficients were not significant in the Right Superior Cerebellar Peduncle (SCP) for HNT, and the Left SCP for RNT. * $p < .05$. ** $p < .01$. *** $p < .001$
